# Isolation of Ontario aquatic bird bornavirus 1 and characterization of its replication in immortalized avian cell lines

**DOI:** 10.1186/s12985-020-1286-6

**Published:** 2020-01-31

**Authors:** Phuc H. Pham, Alexander Leacy, Li Deng, Éva Nagy, Leonardo Susta

**Affiliations:** 1 Department of Pathobiology, Ontario Veterinary College, University of Guelph, Guelph, ON Canada; 2 Department of Pathology and Molecular Medicine, McMaster University, Hamilton, ON Canada

**Keywords:** Avian bornavirus, Aquatic bird bornavirus-1, ABBV-1 replication in avian cells, Persistent infection, Immortalized avian cell lines, duck embryo fibroblasts

## Abstract

**Background:**

Aquatic bird bornavirus 1 (ABBV-1) has been associated with neurological diseases in wild waterfowls. In Canada, presence of ABBV-1 was demonstrated by RT-qPCR and immunohistochemistry in tissues of waterfowls with history of neurological disease and inflammation of the central and peripheral nervous tissue, although causation has not been proven by pathogenesis experiments, yet. To date, in vitro characterization of ABBV-1 is limited to isolation in primary duck embryo fibroblasts. The objectives of this study were to describe isolation of ABBV-1 in primary duck embryonic fibroblasts (DEF), and characterize replication in DEF and three immortalized avian fibroblast cell lines (duck CCL-141, quail QT-35, chicken DF-1) in order to evaluate cellular permissivity and identify suitable cell lines for routine virus propagation.

**Methods:**

The virus was sequenced, and phylogenetic analysis performed on a segment of the N gene coding region. Virus spread in cell cultures, viral RNA and protein production, and titres were evaluated at different passages using immunofluorescence, RT-qPCR, western blotting, and tissue culture dose 50% (TCID_50_) assay, respectively.

**Results:**

The isolated ABBV-1 showed 97 and 99% identity to European ABBV-1 isolate AF-168 and North American ABBV-1 isolates 062-CQ and CG-N1489, and could infect and replicate in DEF, CCL-141, QT-35 and DF-1 cultures. Viral RNA was detected in all four cultures with highest levels observed in DEF and CCL-141, moderate in QT-35, and lowest in DF-1. N protein was detected in western blots from infected DEF, CCL-141 and QT-35 at moderate to high levels, but minimally in infected DF-1. Infectious titre was highest in DEF (between approximately 10^5^ to 10^6^ FFU / 10^6^ cells). Regarding immortalized cell lines, CCL-141 showed the highest titre between approximately 10^4^ to 10^5^ FFU / 10^6^ cells. DF-1 produced minimal infectious titre.

**Conclusions:**

This study confirms the presence of ABBV-1 among waterfowl in Canada and reported additional in vitro characterization of this virus in different avian cell lines. ABBV-1 replicated to highest titre in DEF, followed by CCL-141 and QT-35, and poorly in DF-1. Our results showed that CCL-141 can be used instead of DEF for routine ABBV-1 production, if a lower titre is an acceptable trade-off for the simplicity of using immortalized cell line over primary culture.

## Background

The term avian bornavirus encompasses a diverse group of viruses within the genus *Orthobornavirus* and family *Bornaviridae*. There are five recognized species of avian bornavirus: *Passeriform 1 orthobornavirus*, *Passeriform 2 orthobornavirus*, *Psittaciform 1 orthobornavirus*, *Psittaciform 2 orthobornavirus* and *Waterbird 1 orthobornavirus* [1, 2]. The *Waterbird 1 orthobornavirus* species contains two viruses, aquatic bird bornavirus 1 and 2 (ABBV-1 and ABBV-2). ABBV-1 was first identified in a retrospective post-mortem evaluation of Canada geese and trumpeter swans with neurological disease from Southern Ontario, Canada, using RT-PCR and immunohistochemistry (IHC) on archived tissues [3]. Since then, the virus has been detected in wild geese and mute swans [4, 5], as well as emu and gulls presenting with neurological disease [6, 7].

ABBV-1 is an enveloped negative-sense single stranded RNA virus with a genome of approximately 9 kb, which contains six genes encoding for five structural proteins and one non-structural protein, arranged in order from the 3′ to 5′: nucleocapsid (N), non-structural X protein, phosphoprotein (P), matrix (M), glycoprotein (G), and RNA-dependent RNA polymerase (L) [8]. Throughout the infectious cycle, bornaviruses remain highly cell-associated, and rather than causing cell lysis, infection is long-lasting and persistent [9–13]. Bornaviruses achieve persistence by association of the viral ribonucleoprotein (RNP) with the core histone proteins and chromosomes in the nucleus [14], allowing segregation of viral material between daughter cells without release in the extracellular space and lack of distinct cytopathic effect (CPE). Therefore, visualization of bornaviruses infection in vitro is dependent on indirect methods—frequently immunofluorescence—to detect viral antigens in the absence of CPE [15].

The isolation of viruses in cell culture is considered the “gold standard” for virus identification [16], and the propagation of viruses in cell culture aids in the characterization of virus lifecycles and innate host response to infection. While primary cell cultures are recommended for the initial isolation of numerous avian viruses, including avian bornaviruses [17], the continued use of primary cultures for routine virus production presents multiple disadvantages. Most importantly, primary cultures are often heterogeneous and can have phenotypic variations, such as variability in life span before senescence, within and between batches due to the use of different animals each time a culture is made [18, 19]. Additionally, primary cultures require readily available producer animals or eggs, which can lead to increases in time and costs of preparation, and for wild birds, availability of eggs may be seasonally restricted. Eggs may also be naturally contaminated with viruses. For example, avian bornavirus RNA was detected in the yolk of one egg from a Canada goose [20] and eggs of psittacine birds [21–23]. In addition, Payne et al. (2012) reported in a review article that ABBV-1 RNA was detected in duck embryo fibroblasts (DEF) prepared from embryos of commercial Pekin duck eggs [24]. This problem is likely exacerbated when the virus is highly prevalent in the host population, which can be up to 50% for ABBV-1, as assessed by serology, in certain waterfowl populations depending on location [25]. Therefore, use of immortalized cell lines for routine propagation of ABBV-1 lessens the need for primary DEF and alleviate the associated drawbacks.

The current avian bornaviruses invitrome, which is defined as the collection of cell cultures known to support avian bornaviruses replication [26], is listed in Table 1. Overall, several avian bornavirus strains have been isolated using either primary embryonic cultures or immortalized cell lines, and all species contain at least one virus that can replicate in cell culture (e.g., primary or immortalized). None of these viruses were shown to replicate in mammalian cell lines except for canary bornavirus-2 (CnBV-2) and estrildid finch bornavirus-1 (EsBV-1) (Table 1), albeit at very low rate in infected cultures (< 1%). Although both ABBV-1 and ABBV-2 have been isolated in DEF [4, 27], to date, there have been no published reports describing propagation of either ABBV-1 or ABBV-2 in immortalized cell lines.

**Table 1:** Replication of avian bornaviruses in cell cultures

Virus	Quail			Chicken		Mammalian			Primary			References
	CEC-32	QM-7	QT-6	DF-1	LMH	VERO	MDCK	C6	DEF	CEF	QEF	
ABBV-1	n/a	n/a	n/a	n/a	n/a	n/a	n/a	n/a	Yes	n/a	n/a	[4]
ABBV-2	n/a	n/a	n/a	n/a	n/a	n/a	n/a	n/a	Yes	n/a	n/a	[27]
CnBV-1	Yes^a^	Yes	n/a	Yes	Yes	No^b^	n/a	n/a	Yes	n/a	n/a	[28]
CnBV-2	Yes	Yes	n/a	Yes	Yes	Yes	n/a	n/a	Yes	n/a	n/a	[28]
CnBV-3	Yes	Yes	n/a	Yes	Yes	No	n/a	n/a	Yes	n/a	n/a	[28]
EsBV-1	Yes	Yes	n/a	Yes	Yes	Yes	n/a	n/a	Yes	n/a	n/a	[29]
PaBV-1	n/a	n/a	n/a	n/a	n/a	n/a	n/a	n/a	Yes	No	n/a	[30]
PaBV-2	Yes	Yes	Yes	n/a	Yes	No	No	No	Yes	n/a	n/a	[31–34]
PaBV-4	Yes	Yes	Yes	n/a	n/a	n/a	n/a	n/a	Yes	No	n/a	[30, 33, 34]
PaBV-5	n/a	n/a	No	n/a	n/a	n/a	n/a	n/a	Yes	n/a	No	[35]
PaBV-7	Yes	Yes	n/a	Yes	Yes	No	n/a	n/a	Yes	n/a	n/a	[31]

^a^indicates detectable replication in cell line

^b^indicates no detectable replication in cell line

n/a indicates no data available

*DEF* Primary duck embryo fibroblasts, *CEF* Primary chicken embryo fibroblasts, *QEF* Primary quail embryo fibroblasts, *CEC-32* Immortalized quail embryo fibroblasts, *QM-7* Immortalized quail smooth muscle cells (derivative of QT-6), *QT-6* Immortalized quail fibrosarcoma cells (6 to 24 days old bird), *DF-1* Immortalized chicken embryo fibroblasts, *LMH* Immortalized chicken hepatoma cell line, *VERO* Immortalized African green monkey kidney cells, *MDCK* Immortalized Madin-Darby Canine Kidney, *C6* Immortalized rat glial cells

There were two goals of this study. The first was to describe the isolation in DEF and phylogenetic characterization of infectious ABBV-1 derived from the brain of naturally infected Canada goose from Ontario, Canada. The second was to characterize ABBV-1 replication in immortalized avian cell lines in order to identify a suitable cell line to routinely propagate the virus.

## Materials and methods

### Cells

Four cell culture systems were used, one primary and three immortalized cell lines. The primary culture was duck embryonic fibroblasts (DEF) from Pekin duck (*Anas platyrhynchos* domesticus). The three immortalized cell lines were: duck embryonic fibroblasts (CCL-141) [36] and chicken embryonic fibroblasts (DF-1, CRL-12203) [37], both obtained from the American Type Culture Collection (ATCC), and quail fibrosarcoma cell line (QT-35) [38].

For routine propagation, DEF, CCL-141, QT-35 and DF-1 were grown in maintenance media (Dulbecco’s Modified Eagle Medium [DMEM; Corning] supplemented with 10% fetal bovine serum [FBS; Hyclone] and 1% Penicillin-Streptomycin-Amphotericin B [PSA; Hyclone]). For passaging, cells were washed with phosphate buffer saline (PBS; Hyclone) and dissociated with either trypsin-EDTA (Hyclone) diluted to 0.125% in PBS or TryPLE (Gibco). DEF were passaged every two to 3 days and the immortalized cell lines every five to 8 days, at a split ratio of 1:2 or 1:3. All cultures were incubated at 37 °C in an atmosphere of 10% CO_2_. The method of cell propagation was the same also for persistently infected cells with ABBV-1 (see below).

### Isolation and characterization of ABBV-1 in DEF

ABBV-1 was isolated from the brain of an infected Canada goose (*Branta canadensis*), a kind gift from Dr. Dale Smith of the Ontario Veterinary College. Virus isolation was carried out as previously described [17], with minor modifications. Briefly, goose brain was homogenized and 20 μL were used to directly infect DEF (passage 2, approximately 75% confluence) in a 12-well plate (Nunc), to reach a final 1:10 dilution in DMEM with 1% PSA without serum. The inoculum was kept until DEF were ready to be routinely passaged in maintenance media. Mock-infected DEF were incubated with DMEM alone. Initial confirmation of establishment of a persistent infection in DEF was carried out at the Animal Health Laboratory (University of Guelph) by performing RT-qPCR on cell pellets (10^6^ cells), according to published protocols for detection of the ABBV-1 M gene [39]. Persistently infected DEF were routinely cultured as described above. Cell-free virus stock (section Harvesting and titration of ABBV-1 from infected cell cultures) was produced from DEF and used to infect CCL-141 cells. All the experiments described below were carried out using a stock derived from CCL-141 cells, unless otherwise noted.

To better characterize ABBV-1 replication in DEF, cells in a 15 cm dish were incubated with an approximate 10^3.26^ FFU in 10% FBS/DMEM. Cultures were then passaged every 5 to 8 days, for a total of 13 passages. At defined passage intervals, some of the cells were harvested for RNA extraction and RT-qPCR (passage 1–7), virus titration (passage 6–8, and 10–12), and protein extraction and western blotting (passage 1–3, 6–8, 11–13).

### Sequencing and phylogenetic analysis of ABBV-1

The comprehensive materials and methods for sequencing and phylogenetic analysis of ABBV-1 is provided in Additional file 1: Materials and Methods section. A brief description is provided as follows. ABBV-1 was sequenced using Sanger sequencing (Advanced Analysis Centre, Genomics Facility, University of Guelph) and assembled using Geneious, version 8.0. Phylogenetic analysis was done using 29 aligned nucleotide sequences of the N gene from representative avian bornavirus strains, using the Neighbor-Joining method [40] in the MEGA7 software [41–43].

### Infection of immortalized cell lines with ABBV-1 and comparison as producer cell lines

Two independent infection experiments in immortalized cell lines were performed to characterize the growth and spread of ABBV-1 through cell culture, and establishment of a persistently infected cell populations upon infection. A graphical summary of the experimental outline is reported in Additional file 1: Figure S1.

Briefly, uninfected confluent CCL-141, QT-35 and DF-1 were infected with ABBV-1 in 10% FBS/DMEM at 10^3.49^ FFU in T25 culture flasks (experiment 1), or 10^3.26^ FFU in 15 cm dishes (experiment 2). Cultures were then passaged every 5 to 8 days, for a total of 13 passages. At defined passage intervals, some of the cells were either added to 12-well or 24-well tissue culture plates (Nunc) to be examined by immunofluorescence staining (passage 1–3), harvested for RNA extraction and RT-qPCR (passage 2–7), virus titration (passage 6–8, and 10–12), and protein extraction and western blotting (passage 1–3, 6–8, 11–13).

### Harvesting and titration of ABBV-1 from infected cell cultures

Cell-free virus from persistently infected cell lines (both primary or immortalized) was harvested using a freeze and thaw method. Briefly, cells were detached from flasks with trypsin and centrifuged at 300 x g for 5 min. After centrifugation, cells were resuspended in 5 mL of 2% FBS/DMEM and subjected to three cycles of freezing (− 80 °C) and thawing. The mixture was centrifuged again at 2000 x g for 5 min to pellet cellular debris. The supernatant was collected and stored at − 80 °C.

Cell-free virus was titred either on the same (homologous) cell line from which it was collected, or the same batch of primary DEF (heterologous), using IFA in a 96-well plate format. Homologous titration was used to determine the capacity of each cell line to produce virus and report titre, and heterologous titration with DEF was used to normalize reporting of viral titre produced in different cell lines. Titres of cell-free virus were determined using the Karber method of 50% tissue culture infectious dose (TCID_50_) assay in 96-well plates, using one of the four cell cultures (DEF, CCL-141, QT-35 and DF-1) depending on the experiment (see below). Ten-fold serial dilutions of virus suspension in 2% FBS/DMEM were added to each well (200 μL per well). Five to 7 days post-infection, immunofluorescence (section Immunofluorescence (IFA)) was performed on the plates, and wells were scored positive or negative for presence of virus by observation under a Zeiss fluorescent microscope (Axio Observer.A1). Viral titres were expressed in TCID_50_ units, and converted to FFU by multiplying TCID_50_ by 0.69 [44]; final titers were reported as FFU / 10^6^ cells, as calculated on the number of cells harvested for virus preparation.

For one of the collected passages of ABBV-1 from immortalized cell lines, a RT-qPCR based variation of the TCID_50_ assay (TCID_50_-RT-qPCR) [45] was used where titration was done on 24-well plates instead of 96-well plates and each well was scored for presence of ABBV-1 using RT-qPCR (section Virus titration by RT-qPCR), instead of immunofluorescence staining. TCID_50_-RT-qPCR titration was used to enhance the sensitivity of detection relative to conventional TCID_50_-IFA.

### Immunofluorescence (IFA)

Immunofluorescence assay (IFA) was conducted to evaluate presence of ABBV-1 in infected cells and single wells in 96-well plate format for assessing TCID_50_. A monospecific antibody (Pacific Immunology) raised in rabbit against a peptide spanning residues 332 to 354 of the ABBV-1 N protein (Cys-KEAQLARYRRREVTRGEDGAHLS) was used at 1:1000 concentration. The secondary antibody was a goat antibody against rabbit IgG conjugated with AlexaFluor488 (Invitrogen) used at 1:1000 dilution. Briefly, supernatant was removed from the plates / wells, cells were washed twice with PBS and fixed with 50% alcohol:acetone mixture for 20 min at -20°C. Cells were then washed twice with PBS and blocked for 1 to 3 h with 5% goat serum diluted in PBS-T (PBS with 0.1% Tween-20). After blocking, cells were incubated with the primary antibody diluted in blocking buffer for 1 to 3 h at room temperature or overnight at 4°C. Cells were then washed three times with PBS-T and finally incubated with the fluorophore-conjugated secondary antibodies at room temperature in a dark chamber for 1 to 3 h. After incubation, cells were washed three times, incubated with DAPI (1.43 mM diluted in PBS) for approximately 5 min and observed under a fluorescent microscope. Uninfected cells were used as a negative control.

### Protein extraction, quantification and western blot analysis

Western blot was conducted to determine the relative amount of viral N protein. Briefly, infected cells were washed with PBS and lysed in radioimmunoprecipitation assay (RIPA) buffer (50 mM Tris-HCl pH 8, 150 mM NaCl, 1% NP-40, 0.5% sodium deoxycholate, 0.1% sodium dodecyl sulfate). After 30 min incubation, the cell lysates were centrifuged at 10,000×g for 15 min at 4 °C. The supernatants were collected, and protein concentrations were determined using the Pierce BCA Protein Assay Kit according to the manufacturer’s instructions.

For denaturing SDS-page and western blots, cell lysates were incubated for 10 min at 95 °C on a hot plate to denature proteins. Proteins (1 μg) were separated by 12% SDS-PAGE at 120 V for 1.5 h, transferred to a PVDF membrane with 1x Towbin buffer at 25 V for 1 h (semi-dry transfer, BioRad). Membranes were then blocked with 5% skim milk in PBS-T at 4 °C overnight. The rest of the procedures were performed as previously described [46] and all wash steps were performed with PBS-T. The primary antibodies were either the anti-N protein monospecific rabbit antibody diluted 1:1000 or mouse monoclonal anti-beta actin diluted 1:1000 (ThermoFisher). The secondary antibodies were either goat anti-rabbit or goat anti-mouse IgG secondary antibodies conjugated to horseradish peroxidase (ThermoFisher); positive signal was detected using a BioRad ChemiDoc MP Imaging System and BioRad Image Lab 6.0.1. software.

### Virus titration by RT-qPCR

RT-qPCR was performed on cell cultures infected with ABBV-1 to quantify viral RNA levels over multiple passages after infection. RNA from samples was extracted with RNeasy Mini Kit (Qiagen) according to manufacture’s protocol. Purified RNA was reverse transcribed and amplified using a Luna Universal Probe one-step RT-qPCR kit (NEB) with primers and probes targeting the ABBV-1 N gene. The primer sequences were: forward (5′-ATG CAC TTG CAC TCT TAG AC-3′), reverse (5′-TCC CCA TAA AAC CTC CCA AC-3′), and probe (5′-6-FAM-CCC TGC CCG CAG AGA GAA ATT CCA T-BHQ-3′). The cycling conditions were as follows: 55 °C for 10 min reverse transcription; 95 °C for 1 min initial denaturation, and 40 cycles of 95 °C for 10 s denaturation and 60 °C combined annealing and extension. Cycle threshold (ct) less than 35 was considered to be positive.

### Statistical analysis

Statistical analysis was performed to compare the mean yield titre of multiple passages between persistently infected immortalized cell lines within each independent infection experiment. Since cell-free virus stocks can be harvested from persistently infected cell lines at any and even multiple combined passages after persistent infection, comparing the mean yield titre of multiple passages (as opposed to only one specific passage) between different cell lines accounts for the variability of titre between passages and more accurately determine the cell line that supports better virus production. This was done to compare persistently infected CCL-141 and QT-35 but not DF-1. DF-1 did not produce measurable titre in all but one passage. The titres from all measurable passages were averaged and means compared using Student’s t-test. Results of homologous and heterologous titration were tested separately. Only measurable titres were included in the statistical analysis and measurable titres from all passages of each cell line is provided in Table 2.

**Table 2:** Infectious titre production of ABBV-1 in immortalized cell lines

Experiment 1	Homologous Titration (Log FFU / 10^6^ cells)				Heterologous Titration (Log FFU / 10^6^ cells)			
Passage	CCL-141 ABBV-1	QT-35 ABBV-1	DF-1 ABBV-1	*P* value	CCL-141 ABBV-1	QT-35 ABBV-1	DF-1 ABBV-1	*P* value
8	5.11	2.68	NT		4.86	2.02	NT	
12	3.98	2.43	NT		4.48	1.93	NT	
13	3.92	3.18	NT		4.67	3.09	NT	
Mean ± SD	4.34 ± 0.67	2.76 ± 0.38	N/A	*	4.67 ± 0.19	2.35 ± 0.65	N/A	**
Experiment 2	Homologous Titration (Log FFU / 10^6^ cells)				Heterologous Titration (Log FFU / 10^6^ cells)			
Passage	CCL-141 ABBV-1	QT-35 ABBV-1	DF-1 ABBV-1	*P* value	CCL-141 ABBV-1	QT-35 ABBV-1	DF-1 ABBV-1	*P* value
6	4.57	1.82	NT		4.49	NT	NT	
7	4.40	NT	NT		4.49	NT	NT	
8	4.49	1.82	NT		4.66	NT	NT	
10	3.32	2.90	NT		3.11	2.49	NT	
11	3.61	2.66	NT		3.36	2.40	NT	
12	2.94	2.74	NT		3.11	2.66	2.06	
Mean ± SD	3.89 ± 0.69	2.39 ± 0.52	N/A	**	3.87 ± 0.75	2.52 ± 0.13	N/A	*

NT – no measurable titre and not included in statistical analysis

N/A – not applicable

The mean is the average of all measurable titre within each experiment for each cell line. The SD is the standard deviation of all measurable titre within each experiment for each cell line. Mean ± SD was not calculated for DF-1 ABBV-1 because there was either no measurable titre or only one measurable titre

* *P* < 0.05, ** *P* < 0.01. P value indicates statistical significance between CCL-141 ABBV-1 and QT-35 ABBV-1. Statistical analysis was not performed on DF-1 ABBV-1 due to lack of measurable titre for all but one passage

## Results

### ABBV-1 isolation and characterization in primary duck fibroblasts

**Fig. 1:**
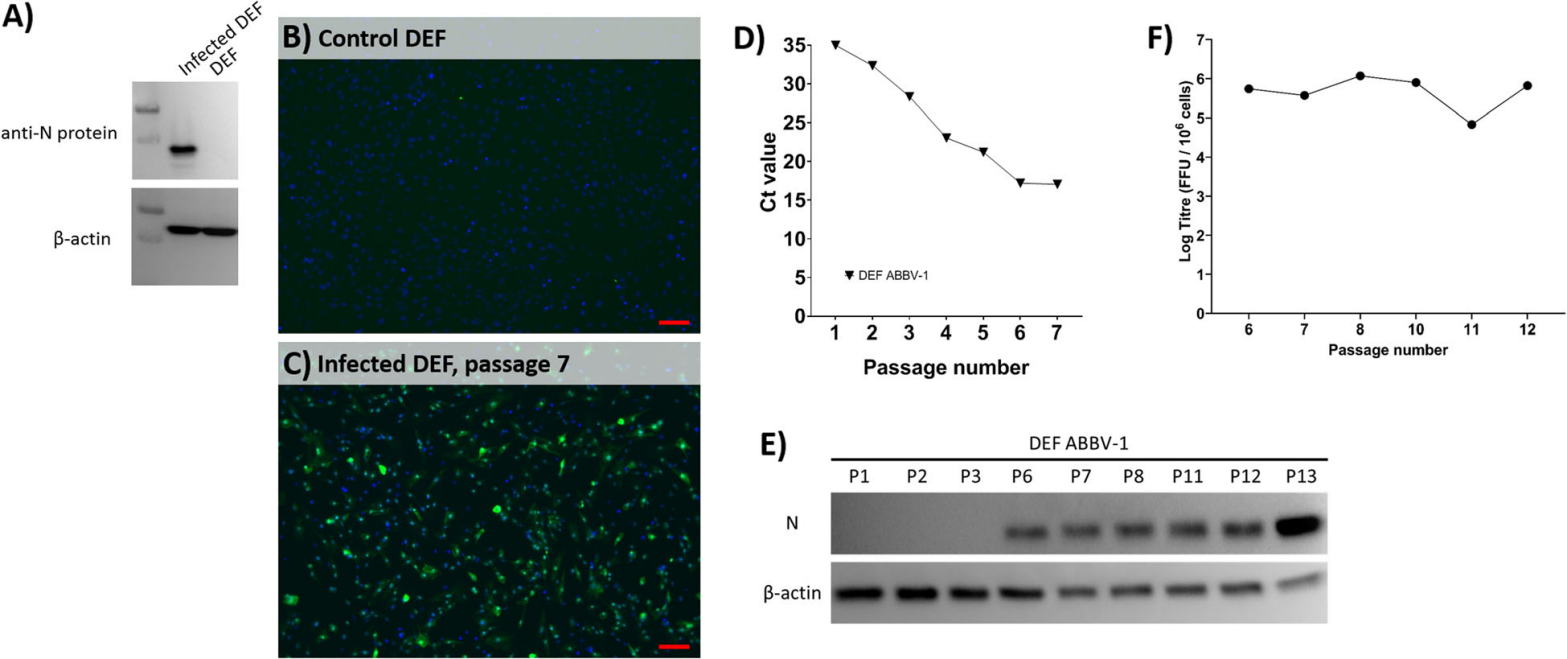
Detection of ABBV-1 from persistently infected DEF using western blot, IFA, RT-qPCR and virus titration. For panels **a**), **b**) and **c**), DEF were incubated with either the brain homogenate from an ABBV-1 positive goose or control medium, and monitored for potential ABBV-1 replication using western blot and IFA. **a** Western blot showing detection of ABBV-1 N protein in lysates from infected DEF but not in uninfected control DEF culture. β-actin was detected in both infected and control DEF cultures. **b** and **c** IFA of control and infected DEF cultures, respectively, using a primary antibody against ABBV-1 N protein (green) and counterstained with DAPI (blue). Scale bar, 100 μm. For panels **d**, **e** and **f**, DEF were infected with cell-culture harvested ABBV-1 and monitored for ABBV-1 replication using RT-qPCR, western blot, and virus titration. D) RT-qPCR of DEF infected with ABBV-1 over the first seven passages. E) Western blot of ABBV-1-infected DEF at passages 1–3, 6–8 and 11–13. F) Virus titration of ABBV-1-infected DEF at passages 6–8 and 10–12

To isolate ABBV-1 for subsequent propagation and evaluation in DEF and immortalized cell lines, DEF were inoculated with homogenized goose brain. At passage 8 and 10 post-infection, presence of ABBV-1 was confirmed by RT-qPCR for the M gene, with Ct values of 19.57 and 17.17, respectively. No virus genetic material was detected in uninfected control DEF. The decrease in Ct values from passage 8 to 10 suggested actively replicating virus. The infectious titre collected from passage 8 to 10 varied between 10^3.85^ to 10^4.72^ FFU / 10^6^ cells. Infection was further confirmed by detecting an approximately 40 kDa protein band in cell lysate (Fig. 1a), and IFA (Fig. 1c) at passage 7 post-infection. Immunoreactivity for ABBV-1 N protein was present in most of the cell population (suggesting establishment of a persistent infection), mainly characterised by a strong coarsely stippled nuclear, and low-level fine cytoplasmic reactivity (Fig. 1c). The isolated virus was designated ABBV-1 UoG-CG.

A second infection experiment was performed on uninfected DEF using new cell culture-harvested virus instead of the original goose brain homogenate. This was done to characterize ABBV-1 replication in DEF better at a known inoculating titre that is not associated with tissue homogenate. Monitoring of the infection over the first seven passages by RT-qPCR showed a steady decline in Ct values over the first five passages, reaching 17.20 and 17.06 Ct by passages 6 and 7, respectively, suggesting beginning of titre stabilization (plateau, Fig. 1d). Western blot of samples collected at passages 1–3 did not detect N protein; however, the corresponding band was visible at passages 6–8 and 11–13 (Fig. 1e). Virus titration performed on cells collected from passages 6–8 and 10–12 ranged between 10^4.83^ and 10^6.07^ FFU / 10^6^ cells (average 10^5.65 ± 0.44^ FFU / 10^6^ cells) (Fig. 1f). Taken together, data from RT-qPCR, titration, western blot and IFA demonstrated that DEF supported the replication of ABBV-1 and could become persistently infected. No cytopathic effect (CPE) was seen.

### Identification and phylogenetic analysis of isolated ABBV-1

The genome of ABBV-1 UoG-CG was sequenced and submitted to GenBank with accession number MK966418. A Basic Local Alignment Search Tool (BLAST) search of the 9006 bp assembled genome sequence showed 97 and 99% identity to European ABBV-1 isolate AF-168 [47] and North American ABBV-1 isolates 062-CQ and CG-N1489 [8], respectively. Phylogenetic analysis with partial N gene sequence that included other 28 avian bornavirus strains showed that ABBV-1 UoG-CG clustered with other ABBV-1 isolates in the *Waterbird 1 orthobornavirus* species (Additional file 1: Figure S2).

### Characterization and comparison of ABBV-1 replication in immortalized cell lines

The replication of ABBV-1 in DF-1, CCL-141, and QT-35 was monitored and compared qualitatively and quantitatively in two independent infection experiments (Additional file 1: Figure S1). Qualitatively, infected cultures at early passages were observed using the phase contrast light microscope to identify possible CPE, and IFA was used to determine spread of the virus in culture. Quantitatively, the efficiency of virus replication in each cell line was measured by two methods: RT-qPCR targeting the N gene to compare viral RNA levels, and TCID_50_ to compare infectious titres. Additional confirmation of ABBV-1 replication was done by western blotting for the N protein. Throughout all the passages, no morphological differences between the infected and uninfected cells were observed under phase contrast light microscopy for any of the cell lines. The other results are described in the sections below.

#### ABBV-1 spread and localization in cell culture

**Fig. 2:**
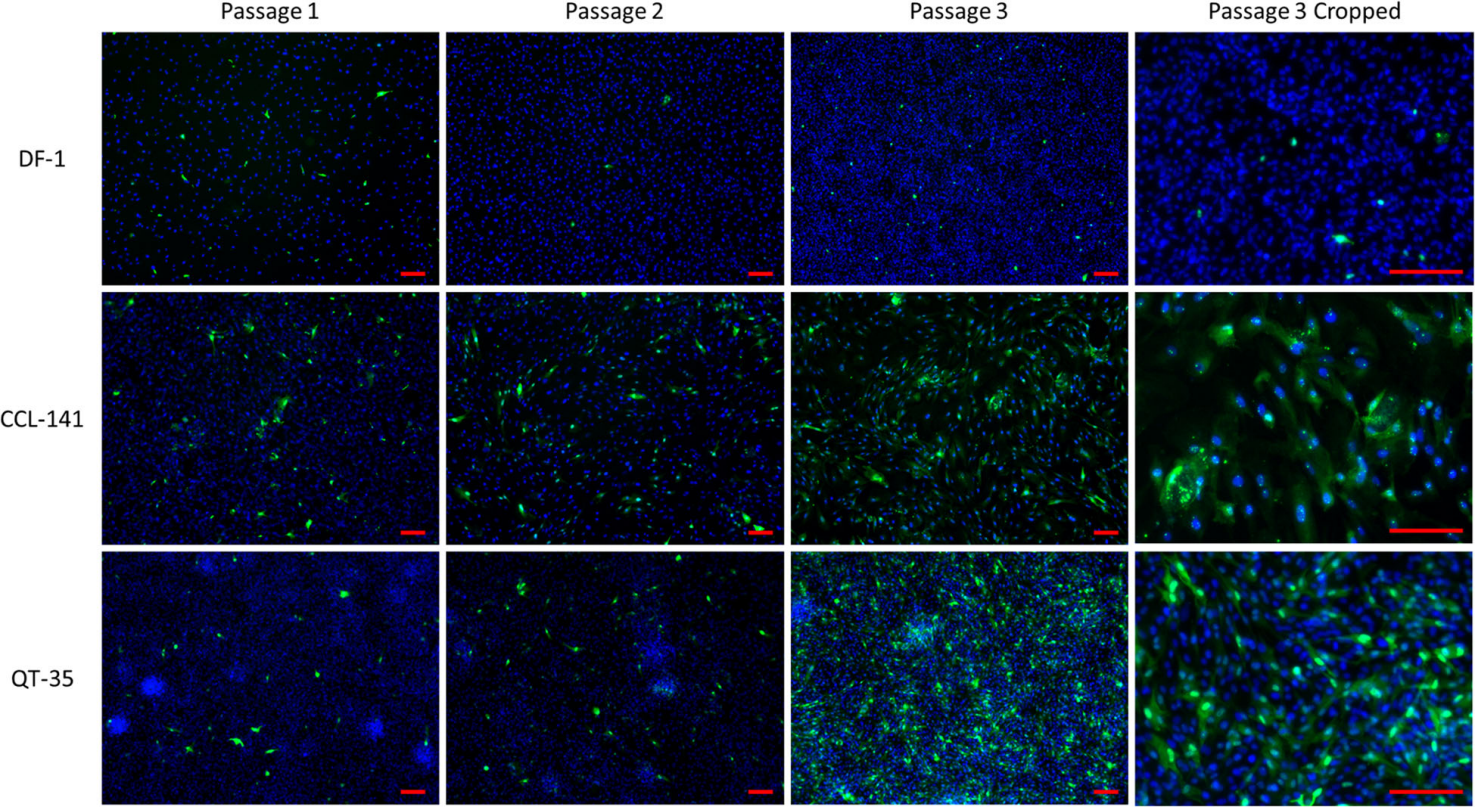
Detection of ABBV-1 replication by IFA, in DF-1, CCL-141, and QT-35 at early passages. Representative pictures of IFA from ABBV-1-infected DF-1, CCL-141, and QT-35 at passages 1–3 post-infection. Blue fluorescence shows DAPI nuclear counterstain, and green fluorescence indicates ABBV-1 N protein. The fourth column shows a magnified area of the picture on the third column. Speckled or punctate signal in the nucleus is more readily observed in infected CCL-141 (fourth column) compared to the than the other cell lines. Scale bar, 100 μm

Early replication and spread of virus in the cell populations were monitored by IFA at passages 1–3 post-infection of experimental infection 1. The time between each passage ranged from 5 to 8 days, and all three cultures were passaged on the same day. In DF-1, there were only rare positive cells for ABBV-1 N protein at all tested passages. In contrast, for CCL-141 and QT-35, the number of infected cells increased over the three passages, with the biggest increase from passage 2 to 3 (Fig. 2). Like what seen in infected DEF, the strongest reactivity for ABBV-1 N protein was seen as nucleus-associated speckled or punctuated signal (Fig. 2).

#### ABBV-1 N gene transcription in cell lines

Viral RNA transcription in cell cultures was monitored by RT-qPCR for up to seven passages post-infection in two independent infection experiments. No viral RNA was detected in uninfected control cells for any line.

**Fig. 3:**
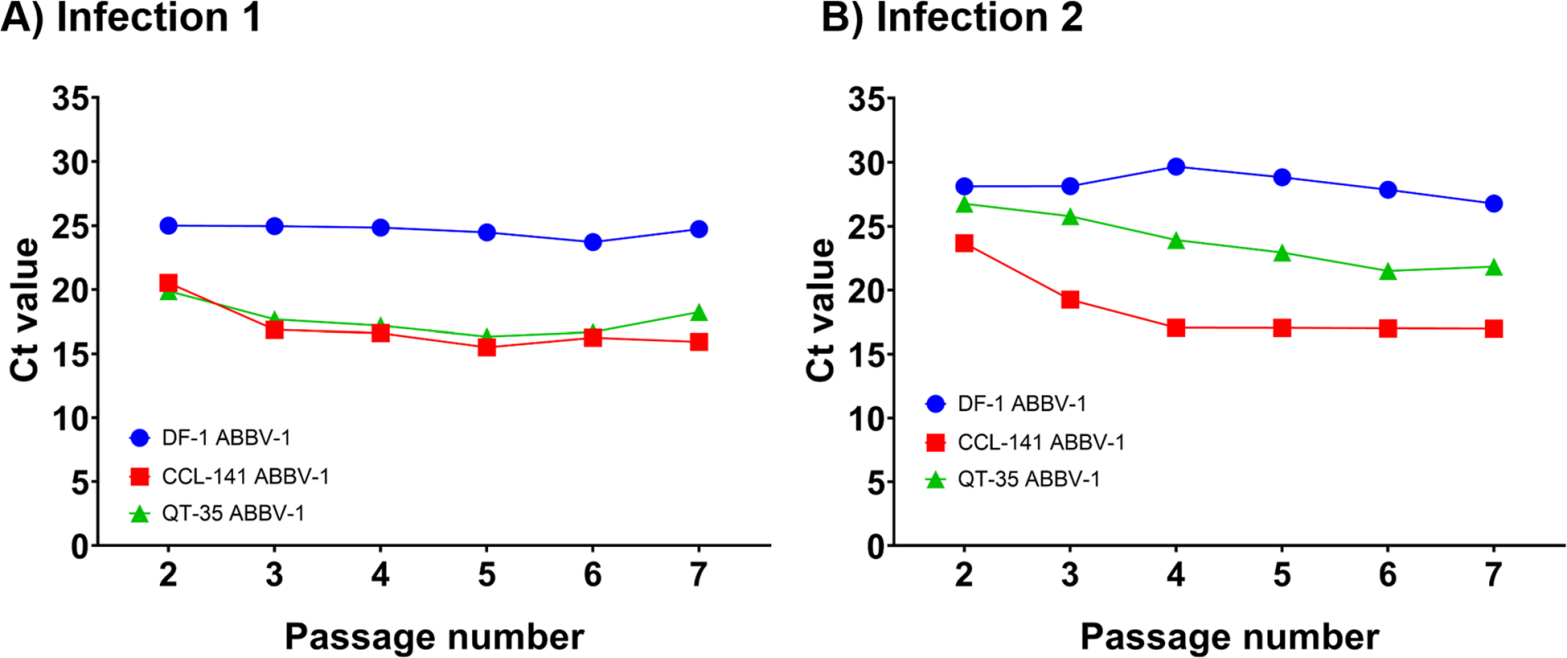
Detection of ABBV-1 N gene, by RT-qPCR, in DF-1, CCL-141, and QT-35 at early passages. RT-qPCR performed on DF-1, CCL-141, and QT-35 infected with ABBV-1 at passages 2 to 7 post-infection in two independent experiments (panels **a** and **b**). The primers and probes targeted ABBV-1 N gene. Cycle threshold (Ct) less than 35 was considered positive, with lower Ct values indicating higher target concentrations. Viral N gene levels increased in CCL-141 and QT-35 with subsequent passages, but in DF-1 remained steady at levels close to the detection threshold

For DF-1, in infection 1, the Ct value was 25.00 at passage 2 and remained between 23.71 and 25.00 over five passages, up to passage 7 (Fig. 3a). In infection 2, the Ct value was 28.12 at passage 2 and remained between 26.77 and 29.67 over five passages, up to passage 7 (Fig. 3b). Both infection experiments suggest that DF-1 can maintain stable, low-level viral RNA transcription.

For CCL-141, in infection 1, the Ct value was 20.52 at passage 2, reached 16.86 at passage 3 and continued to decrease until reaching 15.92 at passage 7, the last measured time point (Fig. 3a). In infection 2, the Ct value was 23.66 at passage 2, reached 19.25 at passage 3 and continued to decrease until reaching 16.97 at passage 7 (Fig. 3b). For both experiments, the biggest drop in Ct value was observed from passage 2 to 3.

For QT-35, in infection 1, the Ct value decreased from 19.88 at passage 2 to 16.33 at passage 5; however, the Ct value increased slightly at passage 6 and 7 (Fig. 3a). In infection 2, the Ct value decreased from 26.76 at passage 2 to 21.83 at passage 7 (Fig. 3b).

#### ABBV-1 N protein expression in cell lines

**Fig. 4:**
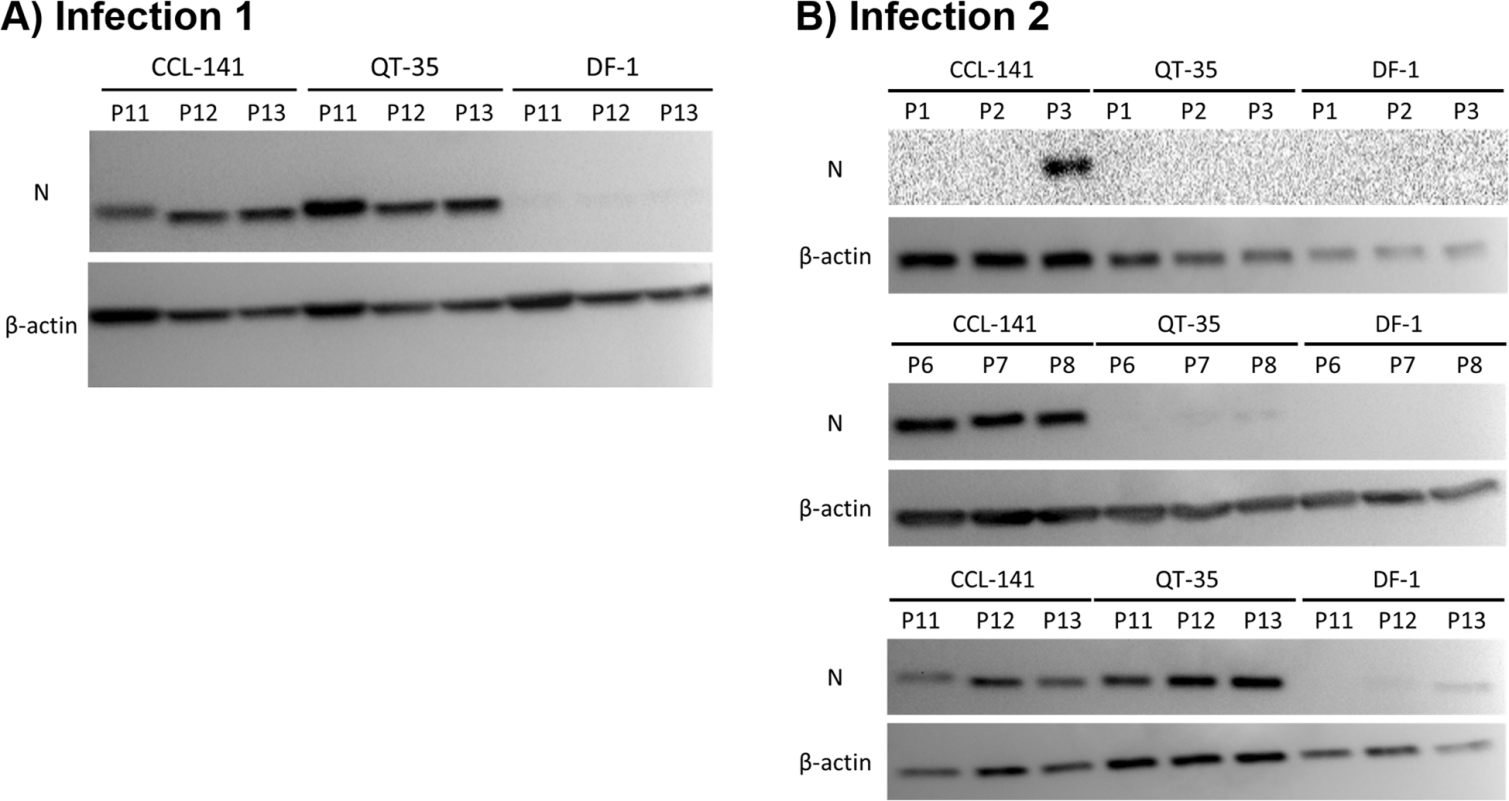
N protein expression in infected DF-1, CCL-141, and QT-35. Western blot of N protein levels in cell lysates collected from cultures of infected DF-1, CCL-141, and QT-35 in two independent experiments. A) Western blot of passages 11–13 (infection 1) showing detection of intense N protein bands in infected CCL-141 and QT-35 cultures and faint N protein bands in infected DF-1 cultures. β-actin protein bands were detected with strong intensity in all samples. B) Western blot of passages 1–3, 6–8, and 11–13 (infection 2). N protein was detected in CCL-141 starting from passage 3, in QT-35 starting from passage 7 (faint band), and in DF-1 starting from passage 12 (faint band)

To examine N protein expression in each cell line, western blots were performed on late passages 11–13 post-infection in infection 1, and on passages 1–3, 6–8 and 11–13 post-infection in the second infection. In infection 1, prominent N protein bands were detected in lysates from infected CCL-141 and QT-35 at late passages 11–13 (Fig. 4a), but only faint bands could be detected from infected DF-1 during the same passages (Fig. 4a). In infection 2, a band corresponding to the N protein was detectable in passage 3 of infected CCL-141 but not in QT-35 or DF-1 (Fig. 4b). By passages 6–8, intense protein bands were detected in infected CCL-141, but only faint or no detectable bands were seen in QT-35 and DF-1, respectively (Fig. 4b). By late passages 11–13, clearly visible protein bands were detected in CCL-141 and QT-35, however only faint bands were visible in DF-1 (Fig. 4b). Taken together, the magnitude of N protein expression appears to be much lower in DF-1 compared to the other immortalized cells lines. For QT-35, N protein expression appeared delayed compared to infected CCL-141. Uninfected cells did not show bands for ABBV-1 N protein.

#### ABBV-1 infectious titre production in immortalized cell lines

Production of infectious ABBV-1 titre in each cell line was measured in two independent infection experiments, and homologous and heterologous viral titre was reported as FFU / 10^6^ cells (see Materials and Methods section).

In infection 1, for CCL-141, titres collected from passages 8, 12, and 13 ranged between 10^3.92^ to 10^5.11^ (homologous titration) and 10^4.48^ to 10^4.86^ (heterologous titration) FFU / 10^6^ cells (Table 2). For QT-35, titres varied between 10^2.43^ to 10^3.18^ (homologous titration) and 10^1.93^ to 10^3.09^ (heterologous titration) FFU / 10^6^ cells (Table 2). For DF-1, according to the results of homologous and heterologous titrations, no measurable infectious titre was detected (Table 2). In the homologous titration, when comparing the mean yield titre between persistently infected CCL-141 and QT-35, infected CCL-141, with 10^4.34 ± 0.67^ FFU / 10^6^ cells, had significantly higher titres than infected QT-35, with 10^2.76 ± 0.38^ FFU / 10^6^ cells (Table 2). The same is true for the heterologous titration with average yield titre of infected CCL-141 at 10^4.67 ± 0.19^ FFU / 10^6^ cells and QT-35 at 10^2.35 ± 0.65^ FFU / 10^6^ cells (Table 2).

A PCR-based variation of the TCID_50_ method (TCID_50_-RT-qPCR) was implemented to increase the sensitivity of the titration assay. This is to confirm the lack of titre in DF-1 is not due to possible low sensitivity of TCID_50_-IFA. The method was completed on passage 8 post-infection for DF-1 and for CCL-141 and QT-35 as positive controls. For DF-1, the titre was once again not detectable, confirming the titration results of the regular TCID_50_. The positive controls, CCL-141 and QT-35, had titres of 10^4.91^ and 10^2.07^ FFU / 10^6^ cells, respectively.

In infection 2, for CCL-141, titres collected from passages 6–8 and 10–12 ranged between 10^2.94^ to 10^4.57^ (homologous titration) and 10^3.11^ to 10^4.66^ (heterologous titration) FFU / 10^6^ cells (Table 2). For QT-35, titres ranged between 10^1.82^ to 10^2.90^ (homologous titration) and 10^2.40^ to 10^2.66^ (heterologous titration) FFU per 10^6^ cells (Table 2); however, passage 7 (homologous titration) and passages 3–6 (heterologous titration) did not yield infectious titre. For DF-1, homologous titration did not detect infectious titre at any passage, while heterologous titration showed detectable titre only in one passage (number 12) out of six, with a titre of 10^2.06^ FFU / 10^6^ cells (Table 2). In the homologous titration, when comparing the mean yield titre between persistently infected CCL-141 and QT-35, infected CCL-141, with 10^3.89 ± 0.69^ FFU / 10^6^ cells, had significantly higher titers than infected QT-35, with 10^2.39 ± 0.52^ FFU / 10^6^ cells) (Table 2). The same is true for the heterologous titration with average yield titre for infected CCL-141 at 10^3.87 ± 0.75^ FFU / 10^6^ cells and QT-35 at 10^2.52 ± 0.13^ FFU / 10^6^ cells (Table 2).

For both infection experiments, when comparing the immortalized cell lines, CCL-141 consistently produced the highest titre range, QT-35 the second highest, and DF-1 produced very low amounts of infectious titre, with only one positive passage. The titre range from immortalized cell lines was lower when compared to the titre range of cell-free virus harvested from infected DEF (10^4.83^ to 10^6.07^ FFU / 10^6^ cells, average 10^5.65 ± 0.44^ FFU / 10^6^ cells, section ABBV-1 isolation and characterization in primary duck fibroblasts).

## Discussions

An ABBV-1 strain (ABBV-1 UoG-CG) from Ontario, Canada was isolated on DEF and characterized in DEF and immortalized avian cell lines: DF-1, CCL-141, and QT-35. DEF were initially used to isolate the virus because it was previously shown to support ABBV-1 replication [4, 48]. The full genome sequence of ABBV-1 UoG-CG was highly similar to two isolates from Texas, U.S.A, with > 99% identity and one from Germany, with > 97% identity. Phylogenetic analysis showed that ABBV-1 UoG-CG clustered with the North American but not European ABBV-1 isolates, suggesting that the Ontario isolate is likely an endemic strain within the North American Canada goose population [47].

ABBV-1 replicated in primary DEF and immortalized cell lines, CCL-141 and QT-35, producing detectable infectious titre. In DF-1, the virus maintained low-level genomic material and protein that was detectable by RT-qPCR and western blot, respectively, but generally did not yield infectious virus particles as determined by TCID_50_ assays. No CPE were observed at any point during passaging of infected cell lines; however, immunofluorescence showed ABBV-1 signal in both the cytoplasm and nucleus, the latter being often more intense. Nuclear-localized replication is a distinctive feature of both avian [27, 49, 50] and mammalian bornaviruses [51–53].

Primary DEF were able to become infected, support replication and yield ABBV-1 infectious particles. This is consistent with what is reported in the literature (Table 1), with numerous avian bornaviruses being propagated in DEF. Some, and presumably most, of the DEF used in those studies [28, 29, 31] derived from Pekin duck (*Anas platyrhynchos* domesticus), as in our case. The titre of ABBV-1 from DEF was in a range from 10^4.83^ to 10^6.07^ FFU / 10^6^ cells in our study; how this titre compares to those in previous publications is unknown, as the ABBV-1 titres from DEF have not been specifically reported [4, 8, 48]. For other avian bornaviruses, PaBV-2 and PaBV-4 in DEF yielded respectively 6.5 × 10^5^ FFU/mL (or 10^5.81^ FFU/mL) [54] and 8.0 × 10^4^ FFU/mL (or 10^4.90^ FFU/mL) [30, 32]. However, these titres cannot be directly compared with our results as PaBV-2 and PaBV-4 were sonicated after freezing and thawing, and the titres were reported as concentrations instead of total FFU. The total volume of these virus concentrations was likely different. Overall, these results showed that embryonic cells from ducks are susceptible to avian bornavirus infections in vitro*.*

Of the three immortalized cell lines examined, CCL-141 [36], also derived from Pekin duck, yielded the highest ABBV-1 infectious titres, although still lower by 1 to 2 logs compared to those derived from DEF. Therefore, CCL-141 appears to be a valid replacement of DEF for routine propagation of ABBV-1, if the simplicity of working with an immortalized cell line over primary culture is desired. For experiments requiring higher titre, DEF is the better choice. To the authors’ knowledge, this is the first report to have tested the ability of immortalized duck cell lines to support replication of any avian bornavirus strain.

The QT-35 quail cell line supported ABBV-1 replication, however, the infectious titre was lower than the one produced from either CCL-141 or DEF. One potential reason for the lower capacity of ABBV-1 to replicate in QT-35 may include the differentiation stages of the cell lines. CCL-141 and DEF were derived from duck embryos [36], while QT-35 from 6 to 24 days old birds [38]. Similarly, other avian bornavirus strains also replicate more efficiently in embryonic cell lines. The immortalized quail embryonic cell line CEC-32 [55] supported better titration of parrot bornavirus (PaBV)-2, 4, and 7 and EsBV-1 from infected tissues than the quail QM-7 myogenic cell line, derived from young birds [29, 31, 33, 56]. Similarly, chicken embryonic cell line DF-1 [37] supported better titration of EsBV-1 from infected tissues than LMH cell line, derived from a 20 days old chicken [29, 57]. Although these difference could simply reflect somatic differentiation, pluripotent embryonic cells typically lack an IFN response [11], suggesting a possible reason for why embryonic cells appear anecdotally more permissive to avian bornavirus replication. The importance of a sound antiviral response in limiting avian bornavirus replication is exemplified by the suppression of PaBV-2 and PaBV-4 replication in CEC-32 and QM-7 cell lines upon exogenous IFN-α addition in the media [58, 59].

One surprising finding was the observed intensity of the ABBV-1 N protein band in QT-35 at later passages relative to the low titre produced by this cell line in those passages. In CCL-141, visually intense N protein bands were also detected, but the titre was higher than in QT-35. In contrast, in DF-1, the faint N protein detection correlated with the lack of virus titre, which was lower than titre in QT-35. This finding was consistent in both independent infection experiments. For QT-35, it is possible that high levels of N protein, relative to infectious virus production, might have affected viral assembly or genome replication by altering the optimal stoichiometric interactions of other viral proteins or causing N protein aggregation. While these speculations need to be experimentally verified, altered N protein expression can impair RNP formation and decrease virus titre, as seen with mammalian 1 bornavirus and other negative-sense single-stranded RNA viruses [60–63].

DF-1 consistently supported low level ABBV-1 gene and protein expression, but no detectable infectious virions up to the 13 passages examined, with one exception. Data in the literature suggest that chicken cells do not support avian bornavirus replication as well as duck or quail cells. For example, PaBV-1 and PaBV-4 did not replicate in chicken embryo fibroblasts but replicated in DEF [30]. Replication of canary bornavirus (CnBV)-1, − 2, − 3, PaBV-2, − 4, and − 7 in either DF-1 or LMH cultures, or both, were lower than in quail cell lines as reported by number of IFA-positive cells, but with no mentioning of viral titre produced by the chicken cell lines [28, 31, 33]. This trend is confirmed in this report with DF-1. However, maintenance of constant low amounts of viral RNA and N protein levels were recorded over multiple passages. This suggests that DF-1 can become infected by ABBV-1; however, the amount of viral RNA and proteins produced is insufficient to consistently support virion assembly. Alternatively, the number of infectious virions produced is too little to establish detectable infection in the TCID_50_ assay reliably. The general difficulty of avian bornavirus to produce abundant infectious virions in chicken cells may reflect host restriction and explain the lack of reports documenting natural avian bornavirus infection in chickens.

## Conclusions

In summary, a Canadian isolate of ABBV-1 (named ABBV-1 UoG-CG) was isolated in cell culture for the first time and was shown to replicated best in primary DEF compared to other three immortalized avian cell lines (CCL-141, QT-35, DF-1). Of the immortalized cell lines, CCL-141 produced the highest titre and therefore can be used as an alternative to primary embryonic fibroblast cultures for producing ABBV-1, albeit with a lower virus yield. DF-1 could be infected but yielded the lowest infectious titre. By characterizing the infection and replication of ABBV-1 in these cell lines, this research expands the repertoire of known cell culture systems available for the study and propagation of ABBV-1.

## Supplementary information

**Additional file 1: Figure S1.** Schematic diagram of cell cultures infected with ABBV-1 over multiple passages (P). Infected CCL-141, QT-35 and DF-1 were monitored over 13 passages using four different techniques, immunofluorescence, RT-qPCR, titration, and western blot, at the passages specified in the diagram. **Figure S2.** Phylogenetic analysis of partial N gene sequence of ABBV-1 UoG-CG. Neighbor-Joining phylogenetic analysis of ABBV-1 UoG-CG partial N gene sequence (1036 positions segment) with 28 other ABV partial N gene sequences. Bootstrap test with 1000 replicates are shown next to the branches. The optimal tree with the sum of branch length = 1.04980780 is shown. ABBV-1 isolate UoG-CG is indicated by arrow. **Supplementary Materials and Methods**. **Table S1.** Primers used for sequencing ABBV-1 UoG-CG.

## Data Availability

Not applicable.

## Abbreviations

ABBV-1 UoG-CG: Ontario isolate of ABBV-1
ABBV-1: Aquatic bird bornavirus 1
ABBV-2: Aquatic bird bornavirus 2
BCA protein assay: Bicinchoninic acid assay
BLAST: Basic Local Alignment Search Tool
CCL-141: Duck embryonic fibroblasts
CPE: Cytopathic effect
Ct: Cycle threshold
DAPI: 4′,6-diamidino-2-phenylindole
DEF: Duck embryonic fibroblasts
DF-1: Chicken embryonic fibroblasts
DMEM: Dulbecco’s Modified Eagle Medium
EsBV-1: Estrildid finch bornavirus
FBS: Fetal bovine serum
FFU: Focus forming unit
HEPES: 4-(2-hydroxyethyl)-1-piperazineethanesulfonic acid
IFA: Immunofluorescence Assay
IHC: Immunohistochemistry
PaBV: Parrot bornavirus
PBS: Phosphate buffer saline
PBS-T: PBS with 0.1% Tween-20
PSA: Penicillin-Streptomycin-Amphotericin B
PVDF: Polyvinylidene difluoride
QT-35: Quail fibrosarcoma
RIPA: Radioimmunoprecipitation assay
RNP: Viral ribonucleoprotein
RT-PCR: Reverse transcription polymerase chain reaction
RT-qPCR: Reverse transcription real time polymerase chain reaction
SDS-PAGE: Sodium dodecyl sulfate polyacrylamide gel electrophoresis
TCID_50_: Tissue culture infectious dose 50%
