# Supplementary material for: Isolation of Ontario aquatic bird bornavirus 1 and characterization of its replication in immortalized avian cell lines
Source: Virol J. 2020 Jan 31;17:16. doi: 10.1186/s12985-020-1286-6 (PMC6995091; doi:10.1186/s12985-020-1286-6)
Supplement: Supplementary file 1 — Additional file 1: Figure S1. Schematic diagram of cell cultures infected with ABBV-1 over multiple passages (P). Infected CCL-141, QT-35 and DF-1 were monitored over 13 passages using four different techniques, immunofluorescence, RT-qPCR, titration, and western blot, at the passages specified in the diagram. Figure S2. Phylogenetic analysis of partial N gene sequence of ABBV-1 UoG-CG. Neighbor-Joining phylogenetic analysis of ABBV-1 UoG-CG partial N gene sequence (1036 positions segment) with 28 other ABV partial N gene sequences. Bootstrap test with 1000 replicates are shown next to the branches. The optimal tree with the sum of branch length = 1.04980780 is shown. ABBV-1 isolate UoG-CG is indicated by arrow. Supplementary Materials and Methods. Table S1. Primers used for sequencing ABBV-1 UoG-CG. [file 12985_2020_1286_MOESM1_ESM.docx]

**Additional File 1**

**Supplementary Materials**

**
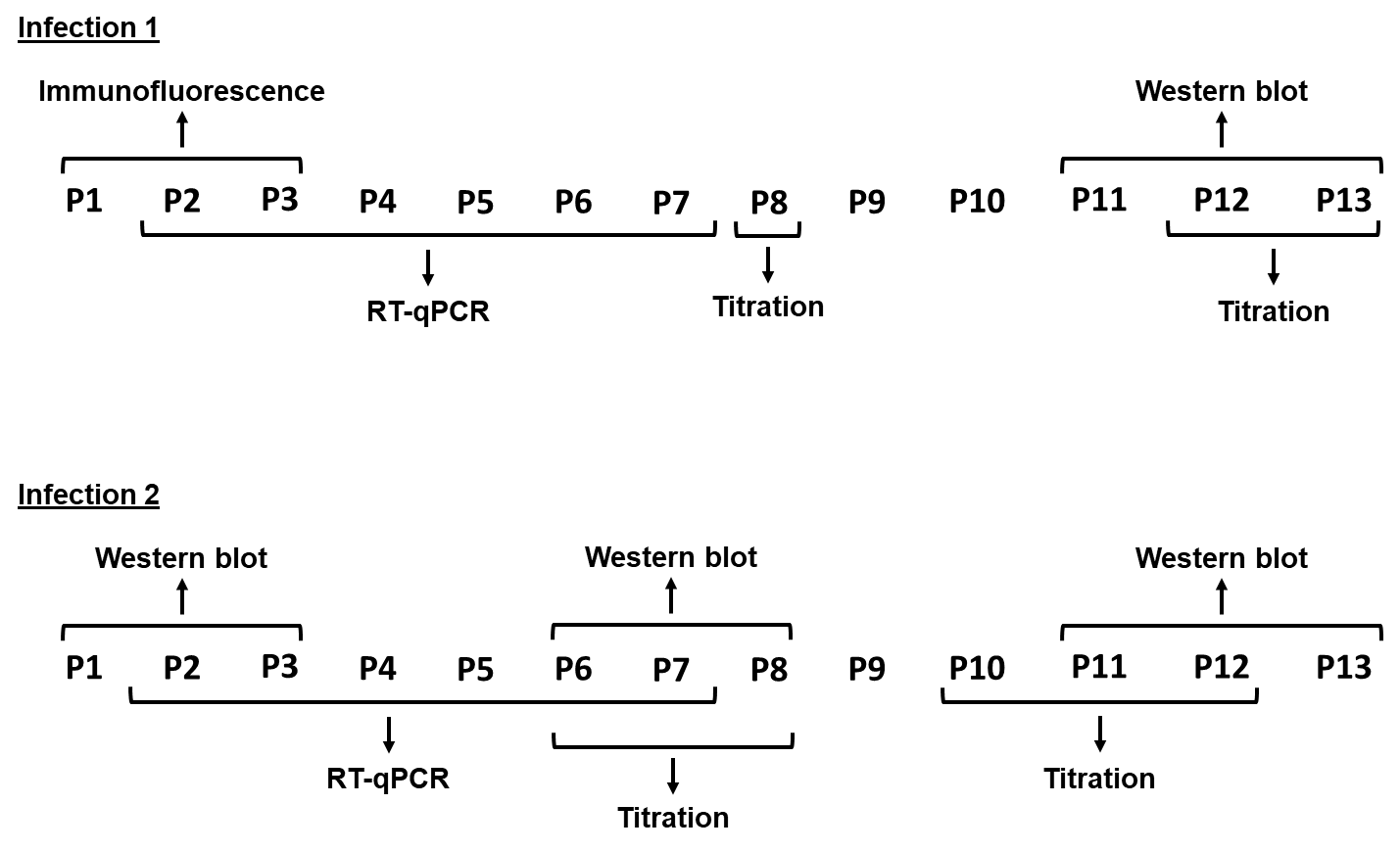
**

**Figure S1. Schematic diagram of cell cultures infected with ABBV-1 over multiple passages (P)**

**
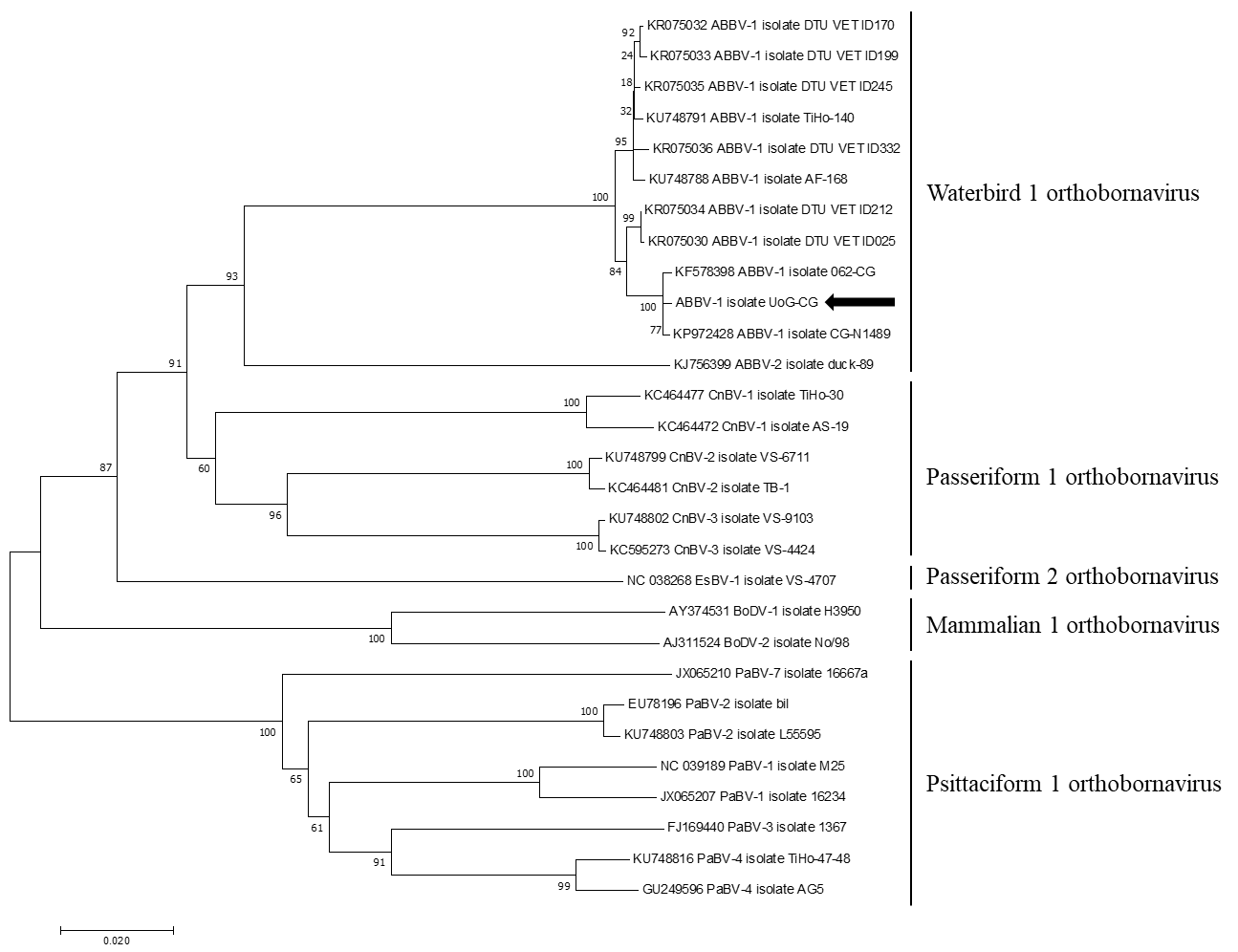
**

**Figure S2. Phylogenetic analysis of partial N gene sequence of ABBV-1 UoG-CG**

**Supplementary Materials and Methods**

**Sequencing of ABBV-1**

The identity of the isolated virus was further confirmed by full-genome sequencing and assembly of the ABBV-1 isolate genome. Based on publicly available ABBV-1 genome sequences from GenBank, a total of 18 overlapping primer pairs (Supplementary Table 1) were used to amplify by RT-PCR the virus genome from nucleotide 80 to 8,957. Briefly, RNA was extracted from DEF pellet using the Roche High Pure RNA Isolation Kit (Roche Diagnostics). Reverse transcription was done using the M-MuLV reverse transcriptase (NEB) and amplicon amplification was carried out using Q5® High-Fidelity DNA Polymerase (NEB), both procedures according to the manufacturer’s instructions. The PCR cycle conditions were: 98 ^o^C for 1 min, followed by 40 cycles of 98 ^o^C for 10 s, 55 ^o^C for 30 s, 72 ^o^C for 75 s, then a final extension of 72 ^o^C for 5 min. Genomic ends were amplified by 3’ and 5’ RACE PCR. The primers for 3’ and 5’ RACE PCR are listed in Supplementary Table 1. 3’ RACE PCR was completed according to a previously published approach (1). Briefly, viral RNA was ligated with adaptor DT88 using T4 RNA ligase (Invitrogen) and cDNA synthesis of ligation product was done using primer DT89. PCR amplification of ligated cDNA was then performed using primers DT89 and Primer 22-R. 5’ RACE PCR was completed using a previously published poly tailing approach (2). Briefly, cDNA synthesis was performed using Primer 19-F followed by poly C-tailing using dCTP and terminal deoxynucleotidyl transferase (TdT; NEB). PCR amplification of c-tailed cDNA was performed using the 5’ RACE AAP and Primer 20-F primers. Amplified genomic regions were resolved on agarose gel, bands were excised, and DNA samples were subjected to Sanger sequencing (Advanced Analysis Centre, Genomics Facility, University of Guelph). Partial sequences were assembled using Geneious, version 8.0.

**Phylogenetic analysis**

Phylogenetic analysis was done using 29 aligned nucleotide sequences of the N gene from representative avian bornavirus strains, using the Neighbor-Joining method (3) in the MEGA7 software (4). Each sequence contained 1036 nucleotide positions. The optimal tree with the sum of branch length = 1.04980780 is shown. The percentage of replicate trees in which the associated taxa clustered together in the 1000 replicates bootstrap test are shown next to the branches (5). The evolutionary distances were computed using the Maximum Composite Likelihood method (6). All positions containing gaps and missing data were removed.

**Table S1.** **Primers used for sequencing ABBV-1 UoG-CG**

| **Name** | **Primer Sequence (5’ to 3’)** | **Genome Position** |
| --- | --- | --- |
| Primer 1-F(2) | TGGACAGCCCAGAAGATATGGAAG | 77 |
| Primer 1-R(2) | TTGTAGTATAAGTAGTCATCTGTGCGT | 775 |
| Primer 2-F(2) | GGAGAAACAGCTACGTTACTACAGA | 589 |
| Primer 2-R(2) | GTCGAGATGCCATTGAGTCTTCTAA | 1286 |
| Primer 3-F(2) | GGCCTGCAACCTTAAACTGAACATA | 1156 |
| Primer 3-R(2) | CGTTGAGGTGGTGGACAAGA | 1824 |
| Primer 4-F(2) | TGATGATGGAGAAGATAGACCTACTTT | 1734 |
| Primer 4-R(2) | CTAACCTCGCATGAAATGACATT | 2419 |
| Primer 5-F(2) | CTCTTAAATGCAACACCGACTCAA | 2320 |
| Primer 5-R(2) | CCTGAACCAAACACACCTAATAAGA | 3094 |
| Primer 6-F(2) | TTTGATGAGTTTAGGCGGTCATATC | 2865 |
| Primer 6-R(2) | CCCAATGGATTGATAAAGGCAAAA | 3628 |
| Primer 7-F(2) | CACGAAACCAATCCGATCAAT | 3483 |
| Primer 7-R(2) | TGTCATTAAACAGCTCTCATTTTCA | 4216 |
| Primer 9-F | CCTCGGAAGGTGGAAGGAATAAA | 3731 |
| Primer 9-R | TCCTTGGACTACAGAAGCAATTAGT | 4291 |
| Primer 10-F | GGACCTAAACGACTGGGAAATTT | 4130 |
| Primer 10-R | GGAGGCCATTTTGCATGTTTT | 4740 |
| Primer 11-F | TGTTGAGAAATCATGGTACTTCCCT | 4558 |
| Primer 11-R | AATCTTCCCTTAACCTTAAGCTCTT | 5157 |
| Primer 12-F | CGTCACGACTTGTTAATGCCTTAAT | 5010 |
| Primer 12-R | TTTCCCAGCAACTTGTGAGAATTG | 5609 |
| Primer 13-F | CACCACATTCATTATGCAAGACA | 5458 |
| Primer 13-R | CAGTGTTGCTGGTGTTGGTAAT | 6118 |
| Primer 14-F | TCATTAACAACCGGGATATGCTTAT | 5978 |
| Primer 14-R | TACATCTTCCAAATGGACTGCCTG | 6577 |
| Primer 15-F | GCCCAACTTTTAGCCAGAGATTTAT | 6410 |
| Primer 15-R | ATTAGGATCTACAACTTTCCGAACCT | 7009 |
| Primer 16-F | CACAGCAATTAAGGTACAGAAGGGT | 6886 |
| Primer 16-R | CCAAGTGAAATACTCGCACTGATAC | 7485 |
| Primer 17-F | ACTTGCTTTGAGAAAATCGAATCTGA | 7325 |
| Primer 17-R | GAGGTCTATATTTGCCTCAGAGACT | 7924 |
| Primer 18-F | TCAAGATTAAACTTTAAAGATCGGAGGG | 7784 |
| Primer 18-R | AAGCAGCACATGTCGGATACATCTA | 8433 |
| Primer 19-F | AATGCTGTTCAAACAAGGGT | 8255 |
| Primer 19 -R | ACACAAGACGTATGATTTTTTAAGA | 8968 |
| DT88 | GAAGAGAAGGTGGAAATGGCGTTTTGG |  |
| DT89 | CCAAAACGCCATTTCCACCTTCTCTTC |  |
| Primer 22-R | GCACAGGAAGATTAAGCTTGG |  |
| 5' RACE AAP | GGCCACGCGTCGACTAGTACGGGGGGGGGGGGGGGG |  |
| Primer 20-F | GCTAGTCAGACAGTGCTTGGT |  |

**References**

1. Li Z, Yu M, Zhang H, Wang H-Y, Wang L-F. Improved rapid amplification of cDNA ends (RACE) for mapping both the 5’ and 3’ terminal sequences of paramyxovirus genomes. J Virol Methods. 2005;130(1–2):154–6.

2. Albuquerque-Silva J, Houard S, Bollen A. Tailing cDNAs with terminal deoxynucleotidyl transferase in RT-PCR assays to identify ribozyme cleavage products. Nucleic Acids Res. 1998;26(13):3314–6.

3. Saitou N, Nei M. The neighbor-joining method: a new method for reconstructing phylogenetic trees. Mol Biol Evol. 1987;4(4):406–25.

4. Kumar S, Stecher G, Tamura K. MEGA7: Molecular Evolutionary Genetics Analysis Version 7.0 for Bigger Datasets. Mol Biol Evol. 2016;33(7):1870–4.

5. Felsenstein J. CONFIDENCE LIMITS ON PHYLOGENIES: AN APPROACH USING THE BOOTSTRAP. Evolution (N Y). 1985;39(4):783–91.

6. Tamura K, Nei M, Kumar S. Prospects for inferring very large phylogenies by using the neighbor-joining method. Proc Natl Acad Sci. 2004;101(30):11030–5.
